# Supplementary material for: Intention to obtain a COVID-19 vaccine among Brazilian immigrant women in the U.S
Source: PLoS One. 2022 Sep 26;17(9):e0274912. doi: 10.1371/journal.pone.0274912 (PMC9512217; doi:10.1371/journal.pone.0274912)
Supplement: Attachment — Submitted filename: Response to Review BWHS COVID Vacc 03.09.22.docx [file pone.0274912.s001.docx]

**Response to Review**

PONE-D-21-22345

Intention to Obtain a COVID-19 Vaccine Among Brazilian Immigrant Women in the U.S.: Results from a Nationwide Survey

**Response to review**

We appreciate the thoughtful comments and questions addressed by the reviewers. We’re confident that the suggestions they have offered will improve the paper. Below, we have responded to all the points raised by each reviewer.

**1. Please ensure that your manuscript meets PLOS ONE's style requirements, including those for file naming. The PLOS ONE style templates can be found at**

<https://journals.plos.org/plosone/s/file?id=wjVg/PLOSOne_formatting_sample_main_body.pdf>

<https://journals.plos.org/plosone/s/file?id=ba62/PLOSOne_formatting_sample_title_authors_affiliations.pdf>

Author response: We believe we have addressed formatting requirements.

2**. Please provide additional details regarding participant consent.** In the Methods section, please ensure that you have specified (1) whether consent was informed and (2) what type you obtained (for instance, written or verbal). If your study included minors, state whether you obtained consent from parents or guardians. If the need for consent was waived by the ethics committee, please include this information.

Author response: Thank you for pointing out this omission. Potential respondents were first required to read and consent to informed consent information before proceeding to the survey. No minors were included in the study, as we note on line 107: “Those eligible to participate were Brazilian women aged 18 years or older...”

We have added the following to the methods (see lines 113-115) to provide additional information about the informed consent process:

“Potential respondents were required to read and consent to study participation before accessing the survey by clicking a box on the survey. Consent information included the purpose of the study, topics addressed by survey questions, and the voluntary nature of participation. The consent language and process were approved by the Institutional Review Board at Tufts University, Medford, MA, USA.”

3.Thank you for stating the following in the **Acknowledgments/ Funding Section** of your manuscript:

“Funding for this study was provided in part by a grant from the Tisch College Community Research Center. Additional funding was provided by the National Center for Advancing Translational Sciences, National Institutes of Health, Award Number UL1TR002544. The content is solely the responsibility of the authors and does not necessarily represent the official views of the NIH.”

We note that you have provided funding information that is not currently declared in your Funding Statement. However, funding information should not appear in the Acknowledgments section or other areas of your manuscript. We will only publish funding information present in the Funding Statement section of the online submission form.

Please remove any funding-related text from the manuscript and let us know how you would like to update your Funding Statement. Currently, your Funding Statement reads as follows:

Please include your amended statements within your cover letter; we will change the online submission form on your behalf.

Author Response: We have removed the funding statement from the manuscript. We note the appropriate funding statement below.

“Funding for this study was provided to JA in part by a grant from the Tisch College Community Research Center (<https://tischcollege.tufts.edu/research/tcrc>). Additional funding was provided by the National Center for Advancing Translational Sciences, National Institutes of Health, Award Number UL1TR002544 (<https://ncats.nih.gov/>). The content is solely the responsibility of the authors and does not necessarily represent the official views of the NIH. The funders had no role in study design, data collection, and analysis, decision to publish, or preparation of the manuscript.”

4. In your **Data Availability statement,** you have not specified where the minimal data set underlying the results described in your manuscript can be found. PLOS defines a study's minimal data set as the underlying data used to reach the conclusions drawn in the manuscript and any additional data required to replicate the reported study findings in their entirety. All PLOS journals require that the **minimal data set be made fully available**. For more information about our data policy, please see http://journals.plos.org/plosone/s/data-availability.

Upon re-submitting your revised manuscript, **please upload your study’s minimal underlying data set as either Supporting Information files or to a stable, public repository and include the relevant URLs, DOIs, or accession numbers within your revised cover letter**. For a list of acceptable repositories, please see http://journals.plos.org/plosone/s/data-availability#loc-recommended-repositories. Any potentially identifying patient information must be fully anonymized. Important: If there are ethical or legal restrictions to sharing your data publicly, please explain these restrictions in detail. Please see our guidelines for more information on what we consider unacceptable restrictions to publicly sharing data: http://journals.plos.org/plosone/s/data-availability#loc-unacceptable-data-access-restrictions. Note that it is not acceptable for the authors to be the sole named individuals responsible for ensuring data access. We will update your Data Availability statement to reflect the information you provide in your cover letter.

Please see the revised Data Availability statement on lines 338-339:

- The dataset for this study can be found at the following link from the Open ICPSR database. <https://doi.org/10.3886/E164382V1>

**Review Comments to the Author**

Please use the space provided to explain your answers to the questions above. You may also include additional comments for the author, including concerns about dual publication, research ethics, or publication ethics. (Please upload your review as an attachment if it exceeds 20,000 characters)

**Reviewer #1:**

1. **On data sharing question/policy: In one place the authors say the data are publicly available, in the other, they say data are not available (see last point in my reviewer comments).**

Author response: Please see the response to #4, above.

Other comments for the authors:
This cross-sectional national online survey of Brazilian women describes attitudes and intentions towards acceptability of a COVID-19 vaccine. While the survey was conducted prior to the emergency approval of the current vaccines, many of the findings may still be useful as the pandemic continues to surge in the face of ongoing vaccine hesitancy. The sample is not representative, but still may provide insights into immigrant attitudes towards the vaccines. **However, the paper could be strengthened by addressing the following points, should the authors choose to revise the manuscript.**

2. **Given the timing of this survey in the context of fast-changing epidemic conditions, I suggest including the dates of the survey in the abstract so readers know right away the referent period for the study.**

Author response: Thank you for this suggestion. The dates of the survey have been added to the abstract. Line 31.

“We conducted an online survey between July and August 2020…”

3. **In the background, some ambiguity exists between the terms “Brazilians” and “immigrant Brazilians.” For example, The authors state there are over 450,000 Brazilians in the US according to Census (L 66). If this is based on the race question, then that may not mean all of these are immigrants. Could the authors clarify? And could they be consistent in use of terms throughout?**

Author response: We have tried to clarify where we are referring to Brazilians born in the U.S. vs Brazilians who immigrated to the U.S. throughout the document. In the introduction, the number we refer to (450,000) does indeed refer to Brazilians born outside of the U.S. We have noted that (see line 63).

4. **Relatedly, Table 1 indicates 11 respondents were born in the US. These cases should be removed from all analyses.**

Author response: Thank you for pointing out this error. Since eligibility criteria for participation included being born in Brazil (see lines 107-108): “Those eligible to participate were Brazilian women aged 18 years or older, were born in Brazil, and reported their current residence as being in the U.S. “Additionally, we have removed those 11 respondents born in the U.S. and re-run all analyses. (Please see Tables 1-3)

5. **Eligibility includes Brazilian women who resided in the US. How did the researchers determine immigrant status?**

Author response: This data was self-reported. On the survey, we inquired about nativity (country of birth) with the question: “What is your country of birth?” with response options including Brazil, United States, or other (specify). Please see lines 143-144.

6. **Most online surveys offering compensation are the target of fraudsters attempting to access the compensation by providing false information. What safeguards were in place to ensure such attempts were blocked?**

Author response: IP addresses of respondents were tracked and checked to ensure that responses were from the U.S. and each individual completed only one survey. While ‘fraud’ is possible, we followed standard procedures used by major survey companies by providing an incentive. We have added a sentence about the possibility of fraud in our survey (see lines 285-287):

“Moreover, since a monetary incentive was provided, we cannot rule out the possibility that surveys were taken by those who were ineligible but misrepresented themselves to gain the incentive.”

7. **Since vaccine hesitancy appears to follow certain geographic boundaries, it may be useful to include geography in the multivariate analysis.**

Author response: It is true that vaccine hesitancy is related to geography. We collected data on the state of residence and have added this information to the methods and results. However, the sample size is not sufficient for us to stratify or analyze for the state of residence.

Lines 146-147

“Moreover, given that vaccine availability differs by geographic region, we asked state of residence”

Lines 191-192

“Most lived in Massachusetts (70.4%), with smaller percentages from New Jersey (6.3%), Florida (5.7%), and California (5.1%).”

**A number of questions arose based on the results/Table 2:**

8. **The selection for the final models is purely empirically driven, not theoretically driven (L 148). Yet it appears the Health Belief Model (or perhaps a related framing) underlies the selection of questions, analysis, and discussion. Are there conceptual framings that may better inform the final models besides just empirical bivariate results?\**

Author response: We had a conceptual model that drove our selection of independent variables and have added this information to the methods section. See lines 88-100.

“The Health Belief Model provides the conceptual underpinnings for this study (19). Specifically, the model predicts that behavior is related to an individual's perceived threat of an illness (perceived susceptibility), beliefs about its consequences (perceived severity), positive benefits and barriers to action, as well as exposure to factors that prompt action (cues to action), and confidence in the ability to succeed (self-efficacy). According to the theory, an individual that perceives a threat to health, is consecutively cued to action and perceives the benefits to action outweigh the barriers are more likely to undertake the recommended preventive health action. In the current study, we conceptualize perceived susceptibility as having had a positive COVID test, perceived severity as subjective perceptions regarding the seriousness of the pandemic (particularly since the information from the Brazilian president suggested otherwise), perceived barriers (e.g., health insurance, limited English language proficiency), and experiencing cues to action through trusted information sources. We also considered factors that have been found in empirical studies to impact beliefs about COVID vaccination, such as age, marital status, household income (20–22), as well as time spent living in the U.S. (23–25).”

9. **The selection of demographic characteristics in Model 1 is confusing. None of them were significantly related to vaccine intentions (Table 1), but somehow the authors landed on Age, marital status and income to include. Why?**

Author response: We have revised the analysis section to clarify how we selected variables for inclusion in the multivariable models. Please see the response to question #8 above and lines 172-176:

“Variables selected for inclusion in multivariable analyses included our key independent variables (prior COVID infection, perceived significance of the pandemic, trusted sources of health information), covariates deemed important predictors of COVID-19 vaccine intentions in prior research (e.g., age, income) (20–22), as well as other sociodemographic characteristics that demonstrated statistically significant associations with vaccine intentions in bivariate analyses (*p* < 0.05).”

10. **Table 2 says that n=364, same as Table 1. However, n=42 cases were missing in the “Trusted Source” variable. What happened to these cases in Model 2? If they were dropped from Model 2, then those same cases should be dropped from Model 1 – otherwise there may be systematic differences in the two samples driving results. Other strategies include imputing missing cases (in general, a better option than dropping cases), or treating them as another category of the variable (similar to what was done for income).**

Author response: Thank you for noting this. You are correct that 42 people didn’t respond to trusted sources of information questions. As a result, we classified these responses as “missing” on that variable, since we did not want to lose information from these respondents from the overall analyses and wanted to use a similar approach as for other variables with missing data (e.g., income). We have added clarification about this process to the analysis section. Please see lines 152-156.

“Additionally, for those missing data for household income and trusted information sources, we categorized these responses as “missing,” and retained these respondents in the sample since we did not want to lose information from these respondents from the overall analyses.”

11. The **interpretation of “Trusted Source” appears to be somewhat off**. Although not explicit on the table (and it should be), the **referent category** for the variable is “Health care provider.” The results, thus, are relative to that category, so that public agencies as the most trusted news source are not significantly different from health care providers for vaccine intention. This interpretation changes the discussion and conclusions. Note – the results themselves may change once the missing data in this variable are appropriate accommodated.

Author response: The interpretation you state is correct. The referenced category is healthcare providers. We elected to use this as the referent since ample evidence suggests that providers are the *most* trusted information sources for COVID-19 vaccines. The interpretation is that respondents did not view public agencies as being significantly different from health care providers as a trusted information source. We have added information to the abstract, results, and discussion to clarify the comparison and to correct the interpretation of results. We have also included clarification in Table 2.

In the abstract (lines 46-47), we state:

“Healthcare providers and public health agencies may be particularly suited to deliver this information given high levels of trust.”

In the results section (lines 228-234), we state:

“Additionally, those who reported that their most trusted sources of health information were social networks (i.e., social media and family or friends) or private news sources (i.e., network news and internet) were significantly more likely to report that they would not be vaccinated compared with those who reported healthcare professionals as their most trusted sources of health information (social networks OR: -1.94, 95% CI: -3.25, -0.63; private news sources OR: -1.71, 95% CI: -2.78, -0.63). Trust in information from public agencies was not statistically different from trust in healthcare providers (OR: 0.10, 95%CI: -1.29, 1.50).”

In the discussion section (lines 306-310), we state:

“An alternative model, also supported by findings in this study, is the dissemination of information through local public health agencies. For example, the use of community health workers that are trained and hired by public health agencies to provide vaccine information has been recommended by the World Health Organization and others (55,56), and this model has been successfully employed in the U.S. (57).”

12. **Finally, the statements about data sharing appear to be in conflict. On the front material, the statement reads data cannot be shared publicly. In the backend topics, the authors declare that data are available on request. Which is it?**

Author response: Please see our response to issue #4 regarding the Data Availability statement.
