# Supplementary material for: Intention to obtain a COVID-19 vaccine among Brazilian immigrant women in the U.S
Source: PLoS One. 2022 Sep 26;17(9):e0274912. doi: 10.1371/journal.pone.0274912 (PMC9512217; doi:10.1371/journal.pone.0274912)
Supplement: Attachment — Submitted filename: Final Response to Review BWHS COVID Vacc 08.18.22.docx [file pone.0274912.s002.docx]

**Response to Review**

PONE-D-21-22345

Intention to Obtain a COVID-19 Vaccine Among Brazilian Immigrant Women in the U.S.: Results from a Nationwide Survey

**Response to review**

We appreciate the thoughtful comments and questions addressed by the reviewers. We’re confident that the suggestions they have offered will improve the paper. Below, we have responded to all the points raised by each reviewer.

**Reviewer #1: The authors have been responsive to comments, and the paper is improved. I have only three comments:**

1. On line 277, the word should be regarding, not regrading

Author response: Thank you for catching this error. We have made the change from “regrading” to “regarding.”

2. On Table 2, the Time in US variable is a bit puzzling. The referent is 0-4 years, but no other category appears for that variable in the table, as one would expect given the categorization of the variable in Table 1. Was this variable dichotomized in the multivariate analysis? Or are the other categories missing from the table? Or was this variable used as a continuous variable, and the reference notation a mistake?

Author response: Thank you for bringing this to our attention. The variable was treated as continuous, and the reference notation in the table was in error. We have corrected this in Tables 1 and 2. Changes to the manuscript text to reflect this correction have also been made (see lines 237-239).

3. The authors describe the HBM as a guiding frame in the Methods, but do not return to it in the Discussion. Assessing the usefulness (or not) of the model for this investigation would be an important theoretical contribution.

Author response: Thank you for this suggestion. We have added language to the discussion section to highlight where our findings were consistent with the Health Belief Model. Additionally, we comment and make recommendations about the utility of the model for investigating COVID-19 vaccine behaviors (see lines 279-280,287-288, 334-345).

**Editor comment**

When you resubmit, please also provide the IRB approval number for the study in the Methods section.

Author response: This has been included (see line 118).
